# A novel revision surgery for treatment of cervical ossification of the posterior longitudinal ligament after initial posterior surgery: preliminary clinical investigation of anterior controllable antidisplacement and fusion

**DOI:** 10.1186/s13018-018-0920-0

**Published:** 2018-08-29

**Authors:** Hai-Dong Li, Qiang-Hua Zhang, Shi-Tong Xing, Ji-Kang Min, Jian-Gang Shi, Xiong-Sheng Chen

**Affiliations:** 1 Department of Spine Surgery, First People’s Hospital affiliated to the Huzhou University Medical College, 158# GuangChang Hou Road, Huzhou, Zhejiang Province China; 2 Department of Spine Surgery, Changzheng Hospital, 415# Fengyang Road, Huangpu District, Shanghai, China

**Keywords:** Cervical, Ossification of the posterior longitudinal ligament, Revision, Antidisplacement

## Abstract

**Background:**

Cervical ossification of the posterior longitudinal ligament (OPLL) is a progressive disease. Posterior decompression surgery is reported to be an effective and comparatively safe procedure with few complications for treatment of patients with myelopathy caused by OPLL. However, some patients require revision surgery because of late neurological deterioration due to OPLL progression or kyphotic changes in cervical alignment. This study reports preliminary clinical results of anterior controllable antidisplacement and fusion (ACAF), a novel revision surgery after initial posterior surgery for OPLL.

**Methods:**

From January 2017 to June 2018, ten patients with cervical OPLL who underwent ACAF revision surgery after initial posterior surgery were included in this study. The mean age was 62.1 ± 8.0 years (52–78), and the mean interval between initial posterior surgery and revision was 78.0 ± 48.2 months (5–180). The Japanese Orthopaedic Association (JOA) scales, Neck Disability Index (NDI), visual analog scale (VAS), and surgical complications were recorded.

**Results:**

The mean surgery time was 179.3 ± 41.8 min (120–240), and the mean blood loss was 432.5 ± 198.3 ml (225–850). The patients were followed up for at least 12 months. The JOA scores improved from 8.7 ± 2.8 to 13.4 ± 2.4; the mean improvement rate was 59.9% ± 16.1%. Postoperative NDI and VAS scores were 13.3 ± 3.7 and 2.0 ± 1.6, respectively, and were significantly improved compared to those before the procedure (*P* < 0.05). Cervical lordosis improved from 3.8 ± 4.3° to 17 ± 4.6° after revision surgery. There was only one instance of cerebrospinal fluid (CSF) leakage; no instances of postoperative hematoma, C5 root palsy, or hoarseness occurred.

**Conclusions:**

The present study demonstrates that excellent postoperative outcomes can be achieved with the ACAF technique for revision treatment of OPLL. Though further study is required to confirm the conclusion, this novel technique has the potential to serve as an alternative surgical technique for revision treatment of OPLL.

## Background

Ossification of the posterior longitudinal ligament (OPLL) is frequently related to cervical myelopathy [1]. Minimally symptomatic patients can be treated conservatively; however, patients with progressive myelopathy require surgical treatment [2, 3]. Several options for treating cervical OPLL have been established involving anterior and posterior surgery. Anterior decompression surgery can directly decompress the cervical spinal cord by removing the ossified ligament and always results in better outcomes and neurological improvement [4, 5]. However, it is considered technically demanding and is associated with serious complications, such as intraoperative neural injury, symptomatic cerebrospinal fluid leakage (CSF), graft dislodgment, and adjacent segment disease [6, 7].

The two typical posterior methods, laminoplasty and laminectomy, are reported as comparatively safe procedures with few complications for the treatment of OPLL [8, 9]. They decompress the spinal cord indirectly, depending on the backward shift of the cervical spinal cord [10]. Long-term outcomes of posterior surgery seem to be favorable, although it is criticized for C5 nerve root palsy, progression of OPLL, and poor cervical lordosis [11, 12]. In cases with poor cervical lordosis or progressive OPLL lesion, neurological improvement is always diminished [8, 13]. Therefore, after cervical posterior surgery, some patients require revision surgery due to late neurological deterioration [14]. Revision surgery is challenging, perhaps because of a combination of progressive kyphosis, segmental instability, massive progressive OPLL, and dural ossification [15].

Sun et al. first described the anterior controllable antidisplacement and fusion (ACAF) technique for the treatment of multilevel severe ossification of the posterior longitudinal ligament with myelopathy [16]. The goal of ACAF is to isolate and “actively transport” residual osteophytes or ossification to restore the space of the spinal canal and thus achieve direct decompression of the neural elements with their location unchanged. The purpose of this study was to investigate the clinical results of ACAF as a revision surgery for cervical OPLL after initial posterior surgery.

## Methods

### Patient population

We conducted a retrospective study of ACAF as a revision surgery performed after initial posterior surgery for cervical myelopathy due to OPLL. From January 2017 to June 2018, ten patients who underwent ACAF revision surgery were identified and included in this study. Follow-up was conducted in all patients for at least 12 months. Patients with severe osteoporosis (WHO criteria), pre-existing spinal deformity, cervical spine trauma, spinal infection, or chronic systemic illness were excluded from the study. This study was approved by the Ethics Committee of the authors’ affiliated institutions, and all the patients signed an informed consent document.

### Radiographic assessment

Based on preoperative radiographic findings, OPLL of the cervical spine was classified into three types: continuous, segmental, or mixed [15]. The K-line connects the midpoints of the spinal canal at C2 and C7 on neutral lateral radiographs. When anterior compression of the OPLL exceeds this line, the K-line is defined as negative [16]. Cervical lordosis was measured as the angle between a line parallel to the posterior aspect of the C2 vertebral body and that of the C7 body. Patients were also evaluated radiographically with plain and dynamic cervical spinal radiographs at 3 and 6 months postoperatively and every 6 months thereafter. Pseudarthrosis was defined as an interspinous motion of > 1 mm on dynamic flexion-extension images.

### Clinical assessment

The Japanese Orthopaedic Association (JOA) scores , visual analog score (VAS), and Neck Disability Index (NDI) were used to measure neck pain, arm pain, and the degree of disability. The improvement rate (IR) of neurologic function was calculated as IR = (postoperative JOA score − preoperative JOA score/17 − preoperative JOA score)/100%. The surgical outcome was defined using the IR as follows: excellent (IR ≥ 75%), good (75% > IR ≥ 50%), fair (50% > IR ≥ 25%), and poor (IR < 25%).

### Surgical technique

**Fig. 1:**
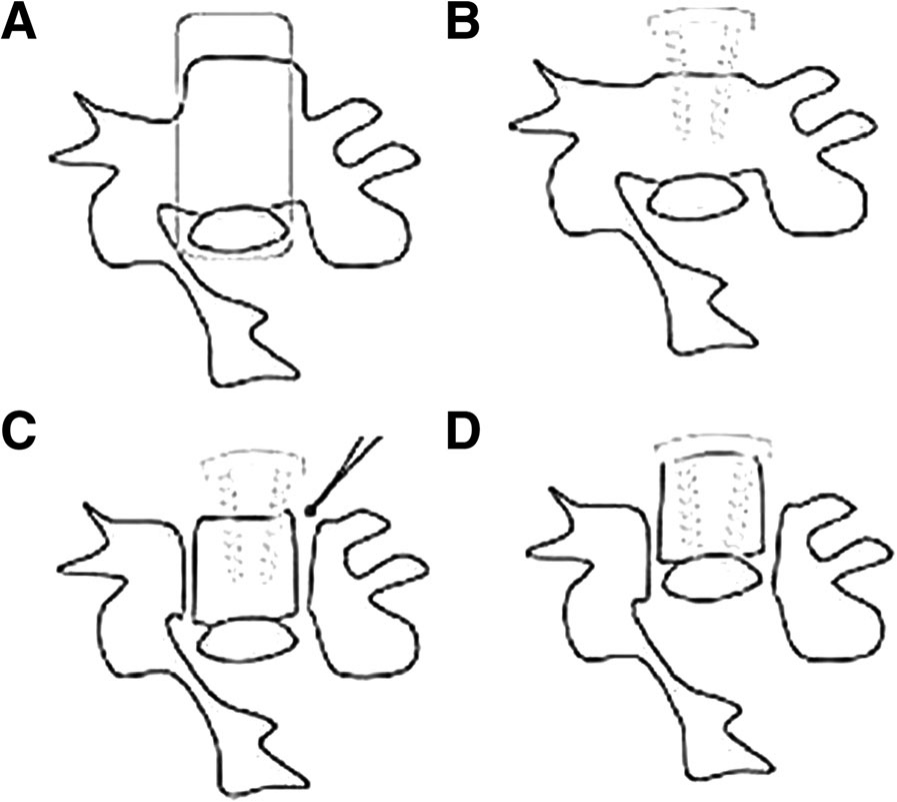
Illustrations of the ACAF technique procedure. **a** The bilateral border of the OPLL mass (dash lines). **b** Installation of the anterior cervical plate (installation of the “bridge”). **c** Bilateral osteotomies of the VOC. **d** Controllable antidisplacement of the VOC by the screws

After general endotracheal anesthesia, the patient was placed in a supine position appropriately padded under the shoulders and neck. Neurophysiologic monitoring, such as somatosensory-evoked potentials (SSEPs), was utilized to predict the neurologic deficit during the operation. The exposure was obtained through a Smith–Robinson approach on the right side. First, discectomies of the involved levels were performed. In the cephalad and caudal levels, the posterior longitudinal ligament was resected to facilitate later hoisting of the vertebrae-OPLL complex (VOC). Then, resection of the anterior vertebral bodies of the VOC was performed using a high-speed burr; third, the intervertebral cages and anterior cervical plate were installed. Fourth, bilateral troughs were created along the widest edge of the OPLL. We used a 2-mm high-speed burr to thin the corticocancellous bone first and 1-mm Kerrison rongeurs to remove the posterior vertebral wall on the bottom of the troughs. Finally, the VOC was hoisted by gradually tightening with screws. An illustration of the procedure is shown in Fig. 1. Autogenous bone pieces were grafted into the bilateral troughs to obtain further fusion. A hard cervical brace was routinely used postoperatively for 3 months.

### Statistical analysis

Statistical analysis was performed using SPSS 16.0. Preoperative and postoperative data such as JOA, VAS, and NDI scores were compared using paired *t* tests. The level of significance was set at *P* < 0.05.

## Results

The patient demographic data are shown in Table 1. According to physical and radiological findings just before revision surgery, we considered the major reasons for neurological deterioration to be anterior spinal cord compression due to residual OPLL progression and local kyphosis.

**Table 1:** Summary of patient demographics and the results of revision ACAF after initial posterior surgery for cervical OPLL

Variable	Value
Sex	
Male	6
Female	4
Age	62.1 ± 8.0 (52–78)
Previous pst op	
Laminectomy	4
Laminoplasty	4
Decompression	2
Mean interval btwn initial op and revision, months	78.0 ± 48.2 (5–180)
Type of the ossification	
Continuous	4
Segmental	3
Mixed	3
K-line	
Minus	6
Plus	4
Mean op time, min	179.3 ± 41.8 (120–240)
Mean blood loss, ml	432.5 ± 198.3 (225–850)
Complications, number of patients	
CSF leakage	1
C5 palsy	0
Postoperative hematoma	0
Implant complication	0

*Pst* posterior, *op* operation, *btwn* between

Values are expressed as the mean ± SD (range)

### Clinical and radiographic results

**Fig. 2:**
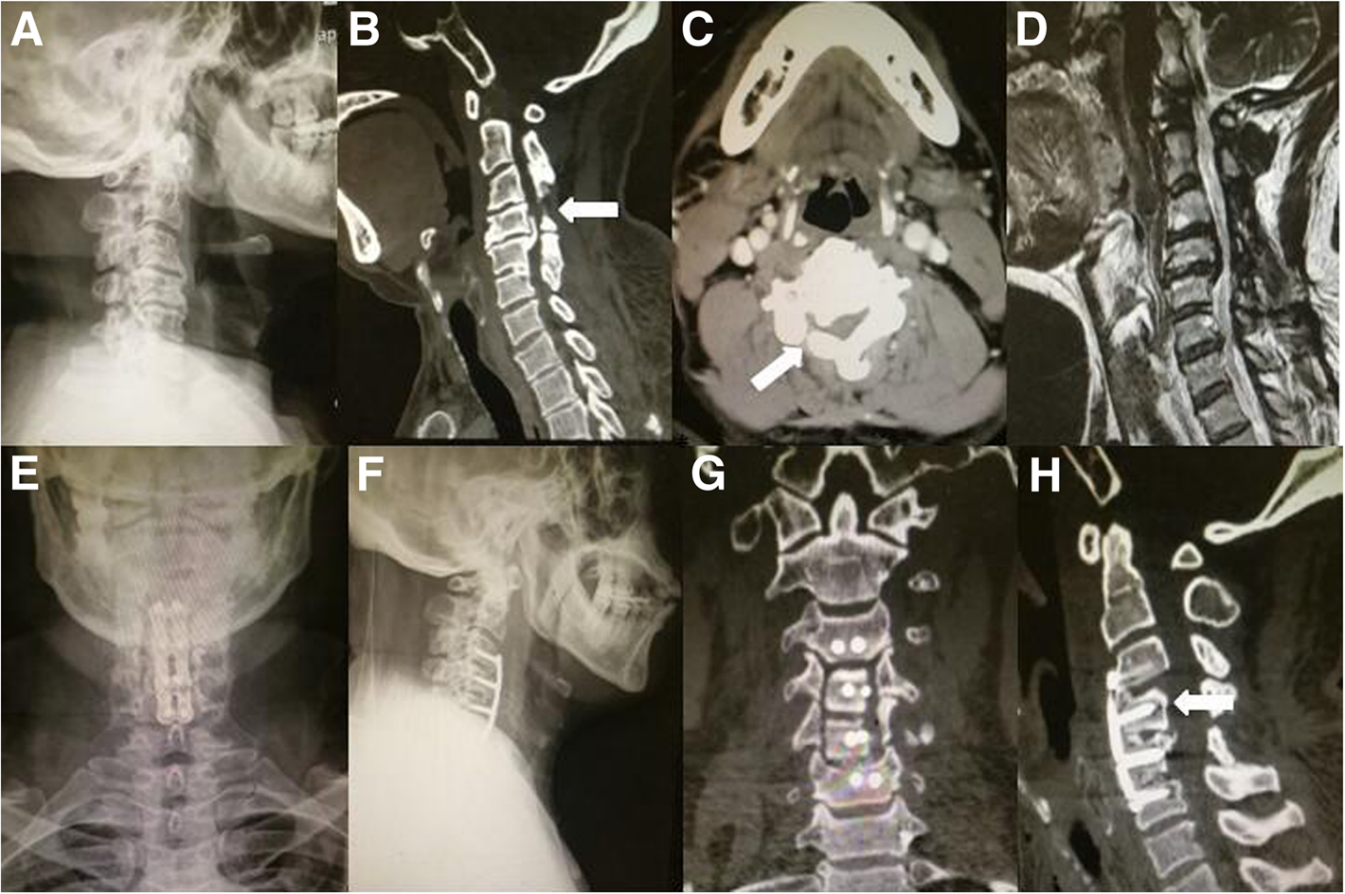
A revision case of a 59-year-old man 5 years after initial posterior decompression surgery. **a** The lateral image showed that cervical kyphosis occurred after the initial posterior surgery. **b**, **c** The CT scan showed that there was only a window decompressing without fixation in the initial posterior surgery (arrows). **d** The MRI showed that the cervical spinal cord of C4–5 was compressed by the OPLL. **e**, **f** The postoperative anterior–posterior and lateral images showed good device positioning and persistent poor cervical lordosis. **g**, **h** The postoperative CT scan showed that the bilateral troughs were created along the widest edge of the OPLL, and we hoisted the VOC by the screws (arrows). We usually used a 2-mm high-speed cutting burr and 1-mm Kerrison rongeurs to remove the posterior vertebral wall on the bottom of the troughs

**Fig. 3:**
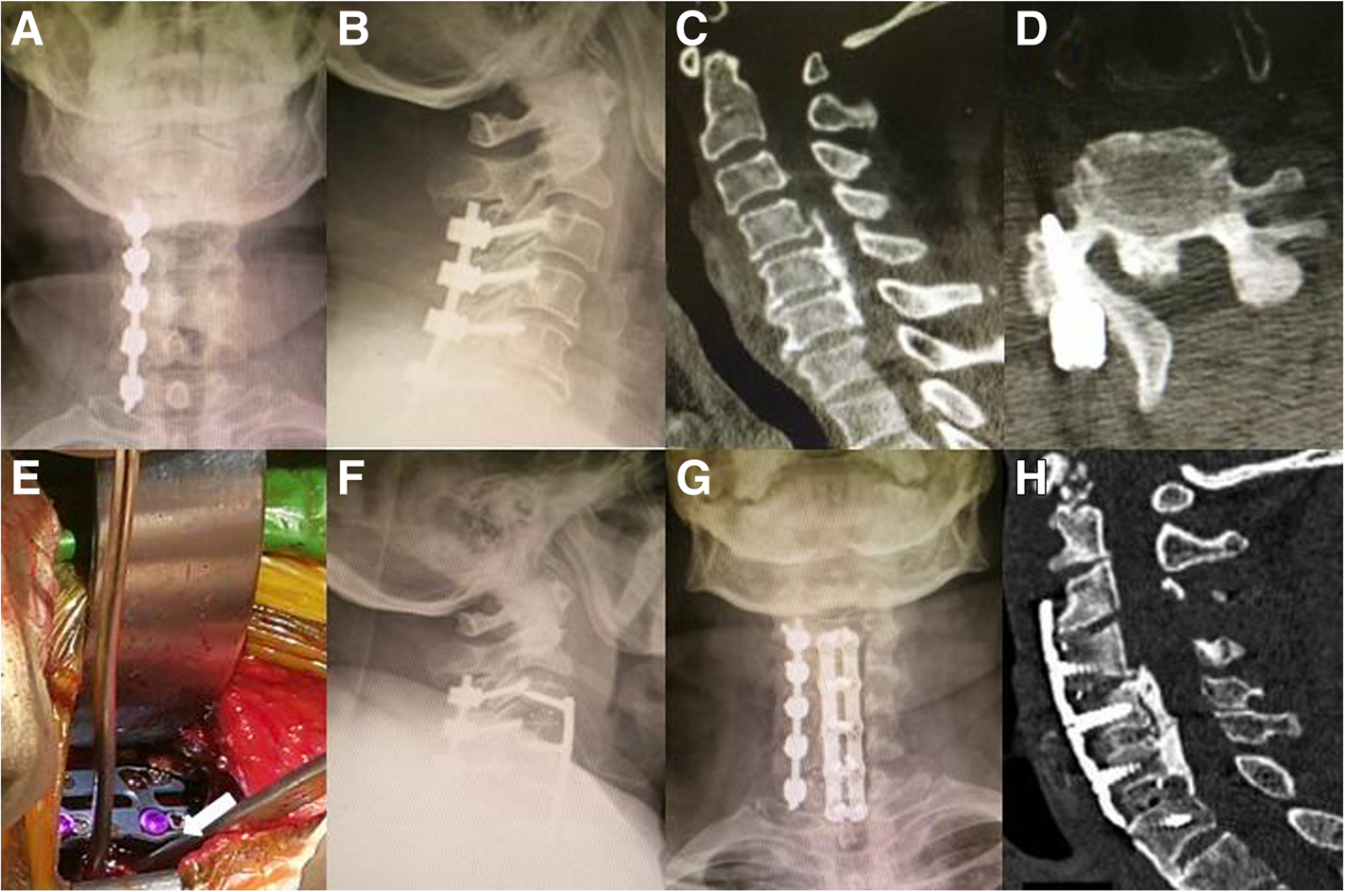
A revision case of a 61-year-old man 12 months after initial posterior laminectomy. **a**, **b** The anterior–posterior and lateral images showed the loss of cervical lordosis after the initial laminectomy. **c**, **d** The CT scan showed that there was a continuous type of OPLL, and only a semilaminectomy with one-sided lateral mass fixation was done in the initial surgery. **e** The intraoperative photo showed that after installation of the intervertebral cages and anterior cervical plate, we used 1-mm Kerrison rongeurs to remove the posterior vertebral wall on the bottom of the troughs for isolation of the VOC (arrows). **f**, **g** The postoperative anterior–posterior and lateral images showed good internal fixation position and improved cervical lordosis. **h** The CT scan showed that the VOC was hoisted forward, and the cervical spinal canal was obviously wider than it was before

The mean operative time in this group was 179 min (range 120–240 min). The mean intraoperative blood loss was 432 ml (range 225–850 ml). The mean JOA score was 8.7 ± 2.8 (range 5–14) preoperatively and 13.4 ± 2.4 (range 9–16) after the ACAF surgery (*P* < 0.05). The average IR was 59.9 ± 16.1%. Two (20%) patients were graded as excellent, six (60%) as good, and two (20%) as fair. The mean VAS score decreased from 4.5 ± 1.6 (range 2–7) preoperatively to 2.0 ± 1.6 (range 0–5) (*P* < 0.05). The NDI decreased from 24.4 ± 10.0 (range 10–40) at the preoperative assessment to 13.3 ± 3.7 (range 8–20) (*P* < 0.05). The postoperative cervical lordosis was 17 ± 4.6°, which was much better than it was before. No patients demonstrated a progression of kyphotic deformity at subsequent follow-ups (all results are shown in Table 2). Images of typical cases are shown in Figs. 2 and 3.

**Table 2:** Clinical and radiological results of patients

Item	Value
JOA	
Before surgery	8.7 ± 2.8 (5–14)
After surgery	13.4 ± 2.4 (9–16)*
NDI	
Before surgery	24.4 ± 10.0 (10–40)
After surgery	13.3 ± 3.7 (8–20)*
VAS	
Before surgery	4.5 ± 1.6 (2–7)
After surgery	2.0 ± 1.6 (0–5)*
Cervical lordosis (°)	
Before surgery	3.8 ± 4.3 (− 7.6 to − 15)
After surgery	17 ± 4.6 (16 to 27)*

*JOA* Japanese Orthopaedic Association scores, *VAS* visual analog score, *NDI* Neck Disability Index

Values are expressed as the mean ± SD (range)

*P < 0.05, compared with the data before surgery

### Complication

There was only one case of intraoperative CSF leakage. The leakage happened when the posterior longitudinal ligament of the cephalad level was resected. Fortunately, it was a small hole and was healed using a sponge and elastic bandage. There were no occurrences of postoperative hematoma or C5 root palsy. No instrument failure was observed during the follow-up.

## Discussion

The gold standard surgical treatment for cervical myelopathy caused by OPLL remains controversial. The choice of surgical method for initial or revision surgery should depend on the location of spinal cord compression, the sagittal alignment of the cervical spine, and the general health status of the patient [17, 18].

The posterior procedure is relatively simple and with low complication rates, and it has been widely used as an initial surgery for cervical OPLL [15]. Though the results of the posterior surgery depend on the backward shift of the cervical spinal cord [5], it has been shown to have the same excellent results as anterior decompression surgery [19, 20]. Relief of symptoms or prevention of symptom progression has been achieved in most patients after surgical decompression of cervical OPLL. However, some patients need revision surgery because of late neurological deterioration [21, 22]. There are two explanations for late deterioration. First, the loss of cervical lordosis or even development of kyphosis is not uncommon after posterior surgery [23, 24]. Second, OPLL tends to progress more often after cervical laminoplasty or laminectomy than it does after anterior decompression surgery [22, 25]. The frequency of OPLL progression has been reported to be as high as 70% [26]. And the mean annual rate of lesion increase was reported as about 3.33% [27].

When considering revision surgery for patients who have already received posterior decompression surgery, it is impossible to again perform decompression from the posterior aspect. Anterior cervical corpectomy and fusion may be a good choice; however, it is considered technically demanding and is associated with serious complications, such as intraoperative spinal cord injury, symptomatic CSF leakage, adjacent segment disease, and graft dislodgment [5]. Macdonald et al. reported that multilevel anterior cervical corpectomy and fusion carries an approximately 22% risk of surgical mortality and morbidity, including pneumonia, deep vein thrombosis, and death [28]. Odate et al. suggested that the use of anterior compression revision surgery for OPLL must be limited due to the high probability of intraoperative CSF leakage and extremely low improvement rate [15]. In their study, surgery-related complications occurred in 63% of patients, the main complication being intraoperative CSF leakage (42%), and the mean improvement rate of the JOA score was only 18%. To minimize the surgical risk of CSF leakage, hemorrhage, and spinal cord injury, Yamaura et al. reported the floating method for the treatment of cervical myelopathy due to OPLL [29]. However, the anterior migration of the OPLL in the floating method is not controlled by the surgeon and owes much to the pressure of the CSF, and approximately 14% of the cases showed inadequate decompression of the spinal canal due to residual ossification with or without postoperative progression of OPLL.

In our study, we used a novel technique called ACAF as the revision surgery for OPLL. It can isolate and actively transport the OPLL ventrally to restore the space of the spinal canal and thus achieve direct decompression of the neural elements with their location unchanged. In contrast to the floating method, the antidisplacement of the OPLL is achieved by the gradual hoisting force of the anterior plate and screws, with immediate feedback. The anatomical basis for the clinical effect of these cases lies in the direct decompression of the spinal cord and nerve roots. Bilateral osteotomies of the vertebrae with a width of 18 mm give enough decompression to the bilateral nerve roots. In this study, the mean improvement rate of the JOA scores was 59.9%. There was only one surgery-related complication (10%) in the study patients. In that case, there was severe adhesion between the dura mater and the ossified posterior longitudinal ligament, and the CSF leak happened when resecting the posterior longitudinal ligament of the cephalad level. Fortunately, it was healed by a sponge and elastic bandage.

### Limitation

This study was only a retrospective study with a small sample size to explore a new revision method for multilevel cervical OPLL after initial posterior surgery. Prospective multiple-center studies, long-term data, and a control group are needed to confirm the result.

## Conclusion

The present study demonstrates that excellent postoperative outcomes can be achieved with the use of ACAF. Though further study is required to confirm the conclusion, this novel technique has the potential to serve as an alternative revision technique for cervical OPLL after initial posterior surgery.
